# Foreign

**Published:** 1841-10-09

**Authors:** 


### FOREIGN.

*Observations on the use of the Vectis, or Single-Blade Extractor, in difficult labours.* By James Borrett, M. D.

(Concluded from page 633.)

In the preceding cases all the women* did well; not an untoward symptom occurred with the exception of the case of J. D., in which flooding supervened. Of the twenty-two vectis cases (in which are two cases of puerperal convulsions) two children were still born; in one only did the death appear clearly to depend on the duration and difficulty of the labour. In the nine forcep cases three children were still born, two being cases of puerperal convulsions.—(See Table, next page.)

*Case* 1.— — Taylor, sixth labour. Her midwife had sent for a surgeon in consequence of flooding, who, before my arrival, had left the woman.

I found her blanched, with a very feeble pulse, and wholly without pains. The os uteri was dilated, the liq. amnii discharged, and the head resting at the brim; the cord prolapsed through the os externum.

As there was no flooding, I tried the experiment of including the mass of cord in a muslin bag, and having carried it above the head, I brought with the vectis the head into the cavity, and in a quarter of an hour she was delivered of a dead child. The loss had been great, and her recovery was protracted.

*Case* 2 — — Knowles was attended by a midwife in her seventh labour, October 25th, 1835. The pains had been throughout regular, and during the last three hours frequent and strong, but did not advance the head. I applied the vectis, and, co-operating with uterine contraction, the child was born with the third pain. Its weight was 9½ lbs. avoirdupois.

*Case* 3.—Mary Bunn, æt. 33: first labour, October 1st, 1831. She sent for the midwife at two P. M. on Wednesday, who did not then remain with her. On Friday, at four P. M., she was again called, and was with her through the night. At seven next morning she left her, and on her return in the forenoon found the os uteri dilated, the liq. amnii discharged, and the pains strong. These continued with regularity, when I was requested to attend at six in the evening. I found that the head came down on the perineum during the pains; the vagina was dry, its orificium unyielding, and the perineum very rigid. During the last seven hours there had been no progress in the labour. I applied the vectis, and in less than half an hour the child was born.

*Case* 4.—On December 20th, 1831, I was requested to visit Sophia Brunton, æt. 22, in her first labour. A midwife had been with her through the night. The os uteri had been fully dilated for eight hours, and the pains regular and of good strength. The head lay in the cavity of the pelvis, with the face to the right side; the right ear was felt behind the symphysis pubis; there was swelling of the soft parts in the pelvis, and of the labia. I applied the vectis, and after repeated powerful co-operation during each uterine contraction, the head was brought through the os externum much elongated, and with a considerable scalp tumour.

*Case* 5.— — Mott, æt. 19, first confinement. Symptoms of labour commenced on Sunday evening. At 3 A. M., she sent for the midwife, who found the us uteri dilated to 2½ inches, and the liq. amnii escaped. At 8 A. M. she sent for me. I learned that the head had scarcely stirred for the last five hours; that the pains, which had been good during the last hour, were less frequent, and broke off short. The vagina was hot, dry, and tender. Having used lard very freely, and awaited a few ineffi-

* I may here observe, that no case of midwifery occurring in my own practice has required more than the ordinary management of women after delivery.

*Table of Difficult Labours terminated by the Vectis from* 1830 *to* 1840, *inclusive.*

| No. | Name. | Age. | Labour. | Remarks. |
|---|---|---|---|---|
| 1 | Taylor | ... | 6th | Accidental flooding—prolapse of cord. |
| 2 | Knowles | ... | 7th | Weight of child (male) 9½ lbs. avoirdupois. |
| 3 | Bunn | 43 | 1st | |
| 4 | Brunton | 22 | 1st | |
| 5 | Mott | 19 | 1st | |
| 6 | Howard | 33 | 8th | Narrow inlet—conjugate diameter. |
| 7 | " | 36 | 10th | |
| 8 | Marshall | 26 | 2d | |
| 9 | Buttifant | 31 | 1st | Child (female) still-born. |
| 10 | Buttifant | 32 | 1st | |
| 11 | Hutchin | 21 | 1st | Weight of child (male) 10lbs. |
| 12 | Spooner | 35 | 7th | Previous labours, five instrumental: in another premature at the 7th month: in one child still-born. |
| 13 | Betts | ... | 6th | Weight of child, 10 lbs.; weight of placenta, 3½ lbs. |
| 14 | Cobb | 33 | 1st | Child died next day; vomited meconium. |
| 15 | Wright | 32 | 4th | Two previous labours instrumental; one by natural efforts. |
| 16 | E. F. | 32 | 1st | Small pelvis, deformed at outlet. |
| 17 | Parker | 37 | 12th | Previous labours, 8 tedious, 3 instrumental; narrow conjugate diameter. |
| 18 | Rushmere | 24 | 1st | |
| 19 | Mansfield | 34 | 6th | Previous labours, 4 tedious; children all small; one at 7th month; weight of child, 7¾ lbs. |
| 20 | Bunn | 35 | 8th | Former labours, 2 instrumental; one at 6th month. |
| 21 | Wright | 39 | 1st | Puerperal convulsions. |
| 22 | Mountain | 24 | 1st | Puerperal convulsions. |

cient pains, I delivered by the vectis; the child still-born.*

*Case* 6.—Elizabeth Howard, æt. 33, of short stature, but healthy and strong, was attended by a midwife in her eighth confinement, September 6th, 1833. None of her former labours had been good, lasting 24 hours and upwards; the child always resting high up. Although the pains had been vigorous and regular for many hours, the labour did not advance. On examination, I found the head thrown forward on the os pubis, the promontory of the sacrum projecting more than is found in a well-formed pelvis; the teguments of the belly were pendulous. The midwife had not, by position or by the binder, endeavoured to obviate the malposition of the head. I passed the vectis, and brought the head to the brim of the pelvis, and aided by steady traction during a few pains it cleared the inlet.

*Case* 7.—In her next labour she was attended by the midwife only. At her 10th labour I was again summoned to her assistance. Having brought down the head into the cavity of the pelvis I withdrew the vectis, and left the termination of the case to the natural efforts. The next was a foot presentation, and in the 12th and last the child was born after thirty hours' hard labour.

*Case* 8.—Marshall, æt. 26, second labour: she became uneasy at three o'clock in the morning of November 8, 1834. The midwife was called to her at 5 A. M. The liq. amnii came away at 11 A. M.: the uterine orifice was dilated to the diameter of 2½ inches. At six P. M. I was requested to visit her; the os uteri was fully dilated: the head had not cleared the brim of the pelvis; the pains had been regular but short and inefficient: she complained of being weary, and in want of sleep. I gave her ♏xx. of laudanum, and left her. At ten o'clock I found she had slept, and that the pains had been renewed, but without effect. I delivered by the vectis.

*Case* 9.—Buttifant, residing in Heigham, æt. 38, first labour, May 28, 1837. I was summonded on Saturday, at 3, A. M., by the midwife, who had been with her since four in the afternoon of the day preceding. My patient began to be uneasy on Tuesday, and passed a restless night, and had little or no sleep on Wednesday and Thursday. Slept in the intervals of pain through the Friday. At 7 P.M. the os uteri was fully dilated, the liq. amnii discharged, the vagina swollen and tender, the pains short and wholly inefficient, the presentation and position natural. I could just reach an ear at the symphysis pubis; the parietal bones were overlapping, the scalp tumor was considerable: she complained much of cramp of the right thigh, and said she was worn out.*

* I did not think the infant died in the birth. From the treatment of her husband, and the appearance of the infant, it is possible it died *in utero.*

* There was very considerable swelling of the whole of the left arm, which followed the removal of a tumor from the breast and axillary margin.

I passed the vectis, and for some time assisted, co-operating with the feeble uterine action, but the head, impacted by the swelling of the soft parts, made no advance. After a brief interval I re-applied the vectis, and at length the head was brought lower down : still the advance was trifling. I again withdrew the instrument, purposing to employ the forceps. The midwife now sat down at the bed-side ; pains followed of rather more strength, but at longer intervals, during which she slept heavilp. On awakening up she complained of thirst; the tongue was dry, pulse slow : the powers of her system were much exhausted, and could not much longer have continued the struggle. I proceeded to apply the forceps, but finding the head much lower I awaited two or three pains ; but as these seemed unequal to accomplish the expulsion of the head, I helped it through the os externum with the vectis : the child still born.

*Case* 10.—Buttifant, living at Trowse, æt. 32, first labour. The midwife was called to her on Sunday, but left, there being no symptoms of labour. She got but little rest, however, that night; the pains increased through the next day, and in the afternoon she summoned her midwife again : the labour proceeded slowly through the night. At six A. M. the os uteri was fully dilated, and the liq. amnii discharged. My assistance was requested by the midwife at 11 A. M.: the pains had been feeble since 6 o'clock, She expressed herself quite worn out: the fœtal pulse was heard : the presentation and position were natural, passages relaxed and dilated, and only efficient pains required to terminate the labour. I watched them for half an hour; they were powerless, scarcely perceptible. Two proceedings were open to me: to secure by an opiate refreshing sleep and recruited powers, or to deliver at once. I preferred the latter course, and shortly delivered her with the vectis.

*Case* 11.—Mary Hutchin, æt. 21 : first labour, July 30, 1833. The pains had been regular and strong during six hours before the midwife requested my assistance. I applied the vectis, and in half an hour she gave birth to a male child weighing 10 lbs.

*Case* 12.—Sarah Spooner, æt. 35, seventh labour, Septembber 12, 1833. She had been delivered with the forceps by surgeons, in five previous labours : in one, she gave birth to a still-born child without assistance ; in another, to an infant at seven months. The midwife had been with her six hours. I found the os uteri dilated to the diameter of two inches, the head resting at the brim. I could not ascertain its position by the hand passed per vaginam : the conjugate diameter was diminished. During the next two hours she vomited often : the pains were frequent and of good strength, but the head did not descend. An hour after the os uteri was dilated ; one-third part of the head had entered, and was fixed in the inlet. Having waited during several powerful pains, which failed to force the head into the cavity, I delivered her by the vectis, co-operating with each labour pain. The child (female) was born with a considerable depression on the left parietal bone.

*Case* 13.—Betts, 6th labour, June 15th, 1835. Had been in strong labour five hours, when I was summoned to her. The os uteri was fully dilated ; the bladder and lower bowel empty. I delivered her quickly by the vectis, of a child (male) weighing 10lbs. ; weight of secundines, 3½lbs.

*Case* 14.—Mrs. C., æt. 38, a strong, healthy person, in her first labour, sent for me, May 8th, 1835. At four A. M., the os uteri, thin and soft, was dilated to two inches, and the liq. amnii discharged ; the pains were quick and strong. At eight A. M. the head was coming down on the perineum, which was firm, dense, and resisting. Warm moist cloths were applied, and lard freely used ; the bowels had been relieved by castor oil. The perinæum not yielding in the least degree during the pains, I bled my patient at two P. M. During the preceding three hours the struggle had been severe, but with a brief pause between the labour throes. I now passed the vectis, and brought the head to bear forcibly during each contraction of the uterus upon the perinæum. At the fourth extractive effort the head suddenly cleared the perinæum, which did not spread out, but rather yielding like a piece of caoutchouc, instantly resumed its dense resistance. The fourchette had not even suffered.

*Case* 15.—Eleanor Wright, æt. 32, fourth labour, November 19th, 1835. Her two first labours were instrumental. In the third a midwife was with her. Her midwife went to her at eight P. M.: the liq. amnii had flowed some hours. At two A. M. (20th) she quitted, the pains having been through the night slow and feeble. At seven A. M. the midwife returned, and at ten my assistance was requested. After waiting three feeble actions of the uterus, I applied the vectis, and brought the head through the os externum. A considerable interval elapsed before the shoulders followed.

*Case* 16.—E. F., æt. 32, of small stature and delicate form : first labour. The midwife went to her on Friday at nine P. M. At ten A. M. on the following day I was summoned by her. The head had not come into the cavity ; I could with difficulty tip the ear; the arch of the pubes admitted only the two fore-fingers. There was an unusual curvature of the sacrum, and the pelvis was small. There was swelling of the soft parts in the pelvis ; scalp tumor considerable ; the pains were good, but they failed to carry down the head. I delivered by the vectis without much difficulty.

*Case* 17.—Eliz. Parker, æt. 37, a tall, strong, and apparently well-formed woman, twelfth labour, October 6th, 1838. She told me all her labours had been slow; that a midwife was with her in the first six, who was detained two and three days together. In her 7th, 8th, and 9th, she was delivered by surgeons with instruments. The midwife who had requested my attendance had been with her twice before. She was called to her at nine A. M., and at five P. M. sent for me. Her report was, the os uteri had been dilated and the pains strong with scarcely an interval of rest during the last four hours, but the labour was not more forward.

On examining, I found the conjugate diameter reduced by the projecting promontorium of the sacrum, and the parietal bones riding. The pains were violent, following each other rapidly, but, as she said, of no use to her. I applied the vectis, and co-operating with four uterine contractions the head cleared the brim, and passed during the next pain through the os externum with the vectis applied, but without any further extractive effort being necessary. The child did not give signs of active life till early the next morning. I found the patient sitting up at the end of a fortnight.

*Case* 18.—M. Rushmere, æt. 24, first labour, February 10th, 1838. At 4 A. M. symptoms of labour commenced, and pains continued through the day. The midwife went to her at 6 P. M. The pains increased in frequency and force as the night advanced, and at 3 A.M. (11th) became very strong. The os uteri had been dilated since midnight. As the labour had made but little progress I was requested to visit her at seven A. M. There was some heat and want of natural secretion; lard was used very freely. I remained with her two hours: the bladder and bowels having acted freely, I left her. On my return at eleven A. M. I found the increase of tumor to proceed from swelling of the scalp: the head was impacted. I immediately delivered her with the vectis.

She suffered from inflammation and discharge from the vagina for ten days after, in consequence of the pressure of the child's head.

*Case* 19.—Sarah Mansfield, æt. 34, sixth labour, July 26th, 1839. In her first confinement the surgeon was at her bed-side for eight hours; the next was premature: her other three children were small, and the labours tedious; she had miscarried once since. The midwife was called to her at four A. M.: the liq. amnii flowed at seven: the os uteri was dilated to two and a half inches, and the pains good, but the head did not descend into the cavity. At noon the uterine action was less powerful, but more prolonged, and without a perfect intermission, the uterus remaining high up in a state of tonic contraction. I was sent for at six P. M. I found the forehead presenting: [the eye was distinctly felt: the os uteri not being fully dilated was compressed between the head and the bones of the pelvis. The conjugate diameter at the brim was narrow. The head was so wedged, that although the parietal bones were riding so as to afford a firm purchase, I could not stir it. There was much swelling of the scalp. She complained of great pain of body, said "she was worn out, and was sure she should die." Having first carried up and secured from pressure the os uteri, I succeeded in bringing down the occiput with the vectis, elevating at the same time the forehead with my left hand, till the anterior fontanelle was to be felt opposite the left sacro-iliac synchondrosis. I now watched the effect of several pains, but there was no advance. She had not felt the child since eleven A. M., and said "it was dead." I had not a stethoscope with me. I now again applied the vectis over the face and chin, and after several pains accomplished the delivery at nine P. M. The child (male) remained in a state of suspended animation for some time. The weight was 7¾lbs. It was much larger than her former children.

*Case* 20.—Bunn, æt. 35, eighth labour. Two of the former were instrumental, one premature.

When I was called to her by the midwife, the os uteri had been dilated, and the liq. amnii discharged five hours. The pains, which had been regular, had become frequent and feeble. I applied the vectis, and after three times co-operating with the uterine contraction, the child was born.

*Case* 21.—Wright, æt. 39; first labour; had miscarried at six months when in India some years before. Her midwife called in the afternoon, and advised her, as she complained of headach, to take a little spiced porter in the evening. She took it, and said she felt better. At 11 o'clock P. M., while preparing to go to bed, she had a convulsive fit. At 1 A. M. I was called in great haste to see her; found her husband and six persons endeavouring to hold her forcibly down during a severe fit. There was no symptom of labour. At 8 A. M. I could with difficulty tip an ear above pubes, the head not having cleared the brim.

I delivered by the vectis: the child still-born. It gave no signs of active life till two hours and a half after its birth. I employed for half an hour the usual means, of bringing about respiration, and requested to have the child warmly covered, except its mouth, and laid carefully aside: two hours after I had left the room the child cried aloud.

*Case* 22.—At 11 o'clock P. M., February 29th, 1836, Mountain, æt. 24, in her first labour, became violently convulsed: the midwife had been with her half an hour. When she got to her the os uteri was dilated, soft, and the vaginal passage relaxed. I delivered her of a living child at 2 A. M.

I have briefly sketched the two preceding cases, as it is my intention in my purposed continuation of midwifery cases to give them more circumstantially.

*Table of Difficult Labours terminated by the Forceps from* 1831 *to* 1840 *inclusive.*

| No. | Name. | Age. | Labour. | Sex. | Remarks. |
|---|---|---|---|---|---|
| 1 | Comar | 37 | 8th | | Three previous instrumental labours. |
| 2 | Peak | 20 | 1st | Female | |
| 3 | Fish | 19 | 1st | Still-born | |
| 4 | Browne | 42 | 8th | Male | Labours all slow; children large. |
| 5 | Davy | 23 | 1st | Female | |
| 6 | Cupper | 39 | 13th | Male | |
| 7 | Rice | 41 | 1st | Female | |
| 8 | Day | 23 | 1st | Male Still-born | Delivery five hours after the first fit; puerp. convulsions. Weight of child nine pounds. |
| 9 | Lynes | 20 | 1st | Female Still-born | Delivered seven hours after first fit; puerp. convulsions. |

*Case* 1.— — Comar, æt. 37, was attended by a midwife, in her eighth labour, Dec. 8, 1832. She had been delivered three times with instruments by different surgeons. At eight P. M. the midwife went to her, and at two in the morning following, my assistance was required; the os uteri was dilated, the liquor amnii discharged; the head had not entered the cavity of pelvis; the pains were regular, but not strong; the head was impeded by a narrow outlet. I returned at nine A. M. There had been no pains during the last two hours. I delivered with the forceps.

Some interesting particulars of adhesion of the placenta over its entire uterine superfices, with contraction of the os uteri, which was overcome with extreme difficulty, I shall take another opportunity of recording.

*Case* 2.—Peak, æt. 20, first labour, October 1st, 1831. She was attended by a midwife, who reported the orifice of the womb had been open, and the waters off for ten hours: the vagina was dry, swollen and tender. I delivered by the forceps. Considerable inflammation of the vagina followed.

*Case* 3.—Fish, æt. 19, was attended by a midwife in her first labour; the head had made no advance for eight hours. I applied the forceps; she was delivered of an infant, male, still-born.

*Case* 4.—Brown, æt. 42, eighth labour, September 13th, 1834. The midwife was called to her at three A. M. Her previous labours had been slow; the children large. I was requested to visit her at five P. M. She had been in strong labour five hours. I delivered with the forceps: child (male) large.

*Case* 5.—J. D., æt. 23, first labour, September 18th, 1836, of delicate frame and of excitable temperament: had suffered much mental anxiety and indisposition of body, with very flatulent and acid stomach and costive bowels. I was sent for at ten A. M. The three previous days she had felt pains, but there was no appearance of labour; she had passed a restless night. The os uteri was thin, soft, and dilated to an inch; the pelvis small, and the arch of the pubes narrow; vaginal secretion scanty. At two P. M. the os uteri was dilated to two inches and a half. At half past six o'clock in the evening the first stage of labour was completed, the os uteri being fully dilated, and the liquor amnii discharged: the head rested high up. During the next four hours the pains were regular and vigorous, but the head made no sensible advance. Acting with the vectis on the occiput I failed to bring down the head, but having carried it round to the pubes, and fixed it over face, ear, and chin, I employed considerable extractive force, and the head descended and was engaged in the cavity of the pelvis. I withdrew the vectis, and awaited the effect of the pains, which followed each other quickly and powerfully, but did not bring the head lower; there was heat and great dryness of vagina; lard was freely used during each pain. I endeavoured, by pressure in front of the ear, to carry the face toward the hollow of the sacrum; the head descended, but seemed as if it would pass toward the anus, and not in the axis of the outlet, the occiput not turning under the pubes. Not being able to accomplish the delivery by manual assistance, I applied the forceps, and at a quarter to twelve delivered the head during the second pain; more than a quarter of an hour elapsed before another pain expelled the shoulders. The child did not cry for some minutes; its face and lips were of deep purple colour. I allowed blood to escape before tying the cord.

A dangerous flooding came on before the exclusion of the placenta, the uterus being emptied and filling again and again, so that I could not leave her bed-side for four hours. It was met by external pressure and cold applications, passing the hand into the uterus, and counterpressure combined, plugging with sponge passed up to os uteri, brandy and laudanum.

*Case* 6.—Cupper, æt. 39, thirteenth labour. She had been poorly through the day, and sent for the midwife at eight in the evening: the labour proceeded, and at midnight the os uteri was fully dilated. At six o'clock A. M., I

was called to assist her; I applied the forceps, and delivered her of a large child.

*Case* 7.—Rice, æt. 41, first labour. She had pains through the day, June 28th, 1837; at eight in the evening sent to her midwife, who, on examination, found the os uteri dilated to an inch, and the liquor amnii escaped; the vagina dry; the pains increased in force and rapidity, so that there was not an interval of five minutes between them. I went to her at eight the next morning; the head lay in the cavity of the pelvis, with the face to the hollow of the right ilium. I touched with difficulty the right ear above the symphysis pubis; the vagina was dry and unyielding, and the os coccygis did not recede on pressure backward. There was much swelling of scalp. During two hours there was no advance of the head, the apparent descent arising from increase of the scalp tumor. I passed with difficulty a blade of the forceps between the head and left ramus pubis; the instrument locked within the vagina, and I succeeded in bringing the head down a little, and then, carrying higher up the blades, which had slipped, I delivered during the fourth pain; child (female) large.

*Case* 8.—On December 16th, 1832, at seven in the evening, I was requested by the midwife to visit Day, æt. 23, attacked with convulsions during her first labour; the os uteri was dilated to two inches and a half. At nine o'clock P. M. she had a second fit: at eleven P. M., I delivered with forceps: child still-born; weight 9 lbs.; weight of secundines 1½ lbs.; there was a narrow conjugate diameter.

I learned that this poor woman died in her next confinement; she had the assistance of surgeons.

*Case* 9.—M. Lynes, October 20th, first labour: was taken with a convulsive fit between two and three of the morning of Thursday, December 4th, 1834. At seven A. M. her midwife went to her, and in half an hour I attended at her urgent request. The os uteri was dilated to two inches. At ten A. M., I again saw her; the forceps was applied, and after considerable difficulty she was delivered of a child still-born.

*London Medical Gazette.*

---

*Substance of a Clinical Lecture on Cancer of Cicatrices, given at St. George's Hospital, May 18th.* By Mr. Cæsar Hawkins.—The subject for to-day's consideration is suggested by the case of a man in Wright's ward, named William Ward, whose limb I amputated three or four weeks ago, as well as by some other cases which have recently been under your notice, as they are instances of a disease that is not commonly understood, and erroneous practice, founded on misunderstanding of its nature, may lead to the cases being trifled with, till it is too late to adopt a successful line of treatment.

This man, Ward, thirty years of age, was admitted into the hospital February 24th, with extensive ulceration reaching from the upper edge of the patella to below the middle of the leg, the surface being full eight inches by five in diameter; in one part, just below the head of the tibia, the ulcer is deeply excavated, from destruction of the anterior part of the bone. A little exposed bone can be felt with the probe, and the leg bends easily at this part; it does not appear, however, that the bone is entirely destroyed, as lateral movement only bends the bone slightly, in comparison with its flexibility in the other direction. The joint does not appear to be at all implicated in the disease. His health has lately declined, and the countenance is pale, but the bowels are regular, and the appetite good; pulse rather quick. He is kept awake by pain, but does not appear to suffer much when the limb is moved or bent.

He states that, twenty years ago, his leg was much injured by being caught in some machinery, after which several pieces of bone came away, but it remained well till the last year, and he was able to use the limb till six months ago, previous to which, ten months before the present time, the skin became ulcerated over the patella, and gradually spread, downwards chiefly, to its present size, the extension below the skin into the bone having only recently taken place. The skin round the ulcer is thickened, and the margin is much raised and hardened, and irregular; and here and there on the ulcer is an imperfect warty appearance, the surface of the ulcer being very red and florid, but being much more irregular and much more condensed than common granulations.

The limb was at first placed in a fracture box, and lettuce and opium given to quiet the pain, and a sedative lotion of prussic acid used, which gave much relief, as it usually does in malignant sores. There being in a few days no doubt of the nature of the case, amputation was proposed to him, but declined.

On the 2nd of April some suspicious enlargement of the glands in the groin took place, but I believe it was only owing to some stimulant having been applied to the sore at one part, the pain from which perhaps contributed to make him at last consent to the only means of cure.

I accordingly amputated the thigh on April 10th, and the stump is now nearly healed, and he has been walking about for some days. The appearance of the disease is seen in the preparations before us; but I will return to that part of the subject presently.

The nature of this case has been described by me in the nineteenth volume of the Medico-Chirurgical Transactions, in a paper in which I have related the results of several cases, the preparations from some of which are on the table before us, together with several others which have occurred since, and which will serve to show you every stage of the disease, which is *cancer* in the imperfect skin of a *cicatrix*.

There takes place, then, an ulceration or morbid growth in old scars of any character, such as those arising from burns, gun-shot wounds, floggings, or common ulcers.

1. Here is a preparation of an ulcer of this character that I took, three or four years ago, from the back of the hand of a man who had had a sore in this situation for twenty years in the cicatrix of a burn, which extended for some distance beyond the ulcer. It was of the size of half a crown, hard and uneven, with a very tender surface and irregular figure, and in part warty in appearance, with a hot prickling or smarting pain running up the arm. Till the last year it had healed from time to time, but since then it has resisted all applications, and has spread. After trying arsenic and other remedies for a little while, I felt satisfied of its nature, and dissected it off from the extensor tendons, which preserved very fair motion after the wound healed. In this case, then, what had been for some time common ulceration at last assumed a new action; and you may see that, although very little warty on the surface, it yet consists of a great number of tough hard fibres, perpendicular to the surface, which may easily be torn asunder; and the tumor is in fact exactly like the hard base of many cases of cancer of the lips.

2. In a further stage, or else anterior to the formation of the hard base of this case, you may see an exuberant growth of firm red warty granulations or fungus, elevated an inch or more above the skin, such as some you may remember in a man named Gale, who was under my care two years ago with a tumour about three inches long, and nearly two broad, situated on the outside of the leg in the cicatrix of an old and large varicose ulcer, the tumor having begun about nine months previously, while the ulcer had first formed eleven years before. The tumor was attached to the fibula, but I believed the bone was not diseased below the periosteum, and that amputation consequently was not necessary. I therefore removed the tumor, and scraped off the surface of the bone with a chisel, and after some trouble, in consequence of the hard cicatrix which extended all round the leg, the wound healed.

3. The disease forms a very considerable sized tumor in some cases, as in these two preparations in which the new growths originated from flogging on the shoulders; or in these preparations from a leg, which I amputated for the disease a few years ago. The tumor in this case extended quite round the leg, and was two inches high, and about four inches broad, and gave excessive pain, having come on a few months before in the cicatrix of a burn, which the patient had had from childhood. Instead of the watery appearance before described, the disease is now, as you may perceive, composed of a firm vascular substance, with round elevations of some size, still capable, to a certain degree, of being torn down in fibres, but bearing more resemblance to a medullary tumor when very vascular. You may see that it only goes down to the fascia and periosteum; even when of this size, the bone, although thickened from inflammation, being still otherwise healthy. This patient at first refused to submit to amputation, but on coming to the hospital one day, I found she would have the operation performed if I would do it immediately; of course I did not refuse, and I saw her some time afterwards without any return of disease.

When I wrote the paper I have alluded to, I expressed my opinion that the disease was one of a malignant nature, but that it was purely a locally malignant disease, as far as I had seen it, and that if it was of the same nature as scirrhous cancer, it was so in the very lowest degree; that it consisted of a new structure, with the power of contaminating the adjoining soft tissues, and converting them into the same disease, and consequently that its entire removal by the knife, or total destruction by caustic, was necessary to effect a cure; but that the low degree in which it was malignant was evinced by a fatal case I had examined after death, in which, although it had spread so as to occupy a space of eighteen inches by ten on the back, it had even then caused no affection of the absorbent glands, nor occasioned the formation of any morbid structure in other parts of the body.

I remember, when this paper was read, that some gentleman present expressed an opinion that the warty ulcer in question was curable; but in fact it is not so; the new growth is incapable, as it would seem, of cicatrising: destroy it as you will to the level of the rest of the skin, yet if any portion whatever of the morbid substance remain, it will show its malignant nature again by fresh growth, as in this beautiful preparation, which I made when I was House-Surgeon here, from a patient whose leg was finally amputated, after all sorts of applications had been tried without success, including several escharotics, and the actual cautery. The peculiar nature of the disease may be sometimes seen in an old ulcer of long standing, in such a manner that only one part of it, or one ulcer out of several on the same limb, may be cancerous, while the rest has the same appearance which it has possessed for years previously.

Rayer, in his excellent work on the Skin, alluding to my paper, or rather to an abstract of it, which he had seen in some journal, supposes the new growth to be an hypertrophy of the papillæ of the skin, whence it derives its warty form; but this is by no means the case, for it affects, as you may see, not the papillæ only, but the whole texture of the cutis, and the preparations show you how different the tumors are from others of common warts, even when very large and numerous, as in this great mass, which I removed from the labium.

Neither is this disease in a cicatrix like the exuberant fungus you may sometimes see, as in this cast, rising out of the cancelli of a bone when it is carious; the fungus granulations in such a case being the result of irritation only, and ceasing when the local condition or the state of the constitution are altered for the better.

Subsequent experience in a good many cases has confirmed me in most of what I then stated, but has shewn me also that the growth of this kind of cancer in cicatrices is more malignant in its influence than I was at that time inclined to believe, and that it bears much resemblance to ordinary cancer of the skin, of which it is evidently a variety, though still it is less virulent than any other variety of cancer which I have seen: its mildness depending probably on its being produced at an earlier period in the imperfect structure of an ulcer than it would be in healthy skin, with a corresponding constitutional tendency to the development of cancer on the application of the necessary excitant, and being therefore also less virulent in its contaminating properties on the parts around, or on the glands or system at large.

Cancer, developed even in original sound skin, instead of a cicatrix, is, however, milder in its usual form than is generally imagined, because we are more familiar with it in other textures, in which its rapid growth and fatal influence are strikingly evinced. Cancer of the skin, for instance, is much less violent than cancer of mucous surfaces, although the two textures are so much alike in their structure and properties. In the penis, or clitoris, or labia, the violent pain, and early influence on the glands, and the frightful sufferings and death of the patient, are very different from cancer, as it usually appears in the skin. You have lately had an excellent example of this in a poor woman named Gaylor, in whom the disease originated in the vagina and labia, and you saw how the glands were enlarged and ulcerated in the groin, and what numerous cancerous tubercles of a secondary form were developed in the skin of the thighs and adjacent parts. It is curious that cancer of the cutaneous structure should be so mild in the majority of cases, since the skin seems to present so active an absorbing surface for various purposes; yet such is doubtless the fact. Here is a preparation of a cancerous tumor, which I removed two years ago from the skin of the sternum in a patient in this hospital, which shows you the same warty appearance of solid texture formed by the disease in common skin, which our other cases present in cicatrices. In this case the tumor was supposed by the man, who was between forty and fifty, to be a common mole, which he observed twenty years before he came here: ten years afterwards it ulcerated, and did not heal again, and within a few months of the operation it began to be painful, and spread more rapidly. He has remained well, I believe, since I removed the diseased part. In another case an elderly man came under my care with a large cancerous mass of warts in the same situation on the sternum, which had ulcerated about four years before, and on his admission was about six inches long, two broad, and one and a half high, with great pain, and an exceedingly fœtid secretion. I wished to have excised it, and to have scraped off the surface of the sternum, which was superficially affected, but he would not consent to it, though I dare say it would have succeeded, as notwithstanding the size of the tumor and its long continuance there was no apparent contamination of the glands in either case. You have recently seen this tumor removed by Mr. Tatum from the back, which was apparently not in a cicatrix, but yet looks like the later stage of cancer in that variety of disease when it forms the large tumor I have spoken of; but unfortunately in this case the glands were much affected, and the patient has died since the operation.

The similarity of the local appearance of the two forms of cancer is therefore very evident; but when I wrote the paper I have referred you to, I had not seen any cases which shewed me, as subsequent experience has done, that there was also so much similarity in their course in other points connected with their malignant properties. First, as to the contamination of adjacent parts. I had seen the disease affect the fascia of periosteum, and then cause enlargement, and thickening, and consolidation of bone, or some degree of ulceration in the cancelli; but it may do more than this, as Ward's case has shown you, and there may be therefore occasionally some difficulty in ascertaining how much disease in any part is really cancerous, and how much is simple inflammation. You have a few days ago seen the thigh amputated by Mr. Babington, and the case is in many respects so instructive that I will venture to speak to you about it, although not under my own care.

Richard Webb, æt. 54, admitted April 28th, with a scirrhous ulcer of the right leg. The ulcer is situated a little below the middle of the leg, over the tibia, irregular on its surface, with several prominent warty granulations; a probe passes freely into a cavity in the bone, which is felt exposed, and in one part destroyed entirely through its substance, so that the probe passes to the soft parts posterior to the tibia. The integuments for some distance around the ulcer are slightly thickened, indurated, and of a purple colour. At present there is not much discharge from the ulcer, but when it becomes profuse it is very offensive. Suffers much from sharp shooting pains up the thigh; a gland in the groin is very slightly enlarged and hardened; superficial veins of leg and foot enlarged. States that twenty-one months ago he struck the part (which is now ulcerated) with a hook: the skin was not broken, and he continued to exercise the limb: it soon became inflamed, and extremely painful, and he expresses having felt a "knot growing from the bone." It continued to increase, and six months after the accident it became ulcerated. Since the ulceration has commenced several spiculæ of bone have come away. Ten weeks ago caustic was applied to it, which impeded its growth, but ineffectually. He is unable to stand. The foot is much benumbed. The tibia appears enlarged in the neighbourhood of the ulcer, and rather irregular to within a short distance of the head. Tongue coated. Pulse quiet. General health good.—States that forty-three years ago he had an ulcer on the same spot from the kick of a horse, which remained open for two years, after which he remained quite well till the blow on the leg.

May 6th.—The leg was amputated above the knee.

Now in this case the cancerous nature of the surface was evident, and with so much disease of the bone of any kind added to the cancer, amputation was necessary, and with the experience of Ward's case just before, it seemed probable to all of us that the bone in this man also was affected with cancer; but then amputation above the knee is much more dangerous than below it, and it was an important question to determine whether there was room to perform the operation with safety against a return of the disease, if the bone itself was cut across below the knee. Our notes remark that the bone was enlarged and irregular in shape and feeling much above the opening into its interior, and it seemed therefore probable to Mr. Keate and myself that the malignant disease was very likely to extend high within the cancelli: Mr. Babington was less afraid, I believe. Nevertheless you will perceive that in fact the operation might have been done below the knee, as the disease in the interior was in all probability only inflammation ending in abscess and necrosis, with enlargement of the shaft; and the cancerous disease was confined, as it would seem, to the cutaneous texture, where the warty and fungous appearance and its vascularity are as well seen now as when the blood circulated in it. The disease, in fact, is only a little further extension of healthy inflammation below the cancerous ulcer, like that which, in the person from whom I removed this limb by amputation, had only caused the deposit of an increased quantity of osseous structure. It is indeed doubtful, when all the man's history is considered, whether the abscess and necrosis did not precede the cancerous action, and whether this did not come on in the skin, after the abscess in the bone ulcerated, the malignity remaining throughout confined to the skin. The disease is in fact, in either supposition, a mixed case: and in another patient, Porter, now in the hospital, with malignant disease of the ankle, you may see that in a cancerous disease beginning below the skin, the ulcer formed in the skin may assume a warty appearance, like that which originates in the cutis.

But now let us return to my own case, that of Ward, and see in our notes and in the preparations what extensive disease may be caused in the bone by cancerous ulceration manifestly commencing in the cicatrix in the skin.

The longitudinal section of the leg shows you how much the skin and subjacent textures are converted into the usual hard fibrous structure of cancer, but you may perceive also at the part where the bone was flexible, that the anterior part of the bone is destroyed, and that the whole cancellous structure is changed into a dense white lardaceous substance, which may be cut with a knife, a few spiculæ only of osseous matter being found in it. The whole head of the bone is changed into this new growth, leaving the cartilage however in its normal condition, and there is no sign of disease in the articulation. You may perceive that the cancelli, as low as where the bone was sawn across about the middle of the leg, have undergone this alteration into a cancerous tumour; in fact, although several inches were allowed below the part where the bone was flexible, yet even lower than this the bone has become diseased, leaving only a few inches from the ankle free from it. In this transverse slice of the lower end of the bone the change is very well seen, as one portion (its posterior surface) is still composed of healthy osseous tissue, while the other two surfaces of the outer shell, and the greater part of the cancelli, are lardaceous and white, and softer than usual, looking like what you may see in a tumour unconnected with bone.

The appearance of the new growth is not, however, like brain; it is not like a medullary or hæmatoid variety of malignant structure, but is much more like the scirrhous form of carcinoma, using this term in its genuine sense.

In this next preparation cancerous disease of the tibia has gone to a further stage in a man whose leg was amputated by Mr. Walker, and you may perceive that besides the infiltration of the cancelli with cancerous matter, and the absorption of the old bone to make way for the new deposit, there has been extensive destruction in several parts by deep ulceration, forming excavations in the interior of the bone, in a much more marked degree than in Ward. There was some doubt whether the disease might not have originated in the bone, and ulcerated outwards into an old cicatrix, the disease then presenting the usual warty growth and fungous appearance of cancer of cicatrices; but if it were so, it equally shows that cancer of a cicatrix is possessed of certain characters, in whatever way it begins, and that both the skin and the bone may be extensively destroyed by ulceration. The patient died of secondary abscesses, I believe, without morbid deposits of cancerous kind in the rest of the body.

*Secondly*, with regard to the absorption of the poison of the cancerous ulcers of cicatrices. When this patient, Ward, was unwilling to submit to amputation, you will remember that we had reason to fear that the glands in the groin were becoming contaminated, but our apprehensions were probably excited by simple irritation and enlargement, without malignant infection.

The correspondence between cancer of cicatrices and that of healthy skin, as to its effects on the glands, had not been witnessed by me when my paper was written, but I have since then had an opportunity of seeing that the absorption does take place, and the case is an example at once of this fact, and of the mildness of the disease, on account of the length of time it had existed before the glands were affected. It happened in one the patients whose cases were detailed in the paper, a man of the name of Callcott, who was operated on by Sir B. Brodie in 1828, the disease appearing to be connected with the tibia, which had been injured twenty-seven years previously, and having then existed fourteen months. A portion of bone was removed by the trephine along with the cancerous fungus, but it was found to be only vascular and inflamed, and not changed into cancer, as in Ward. The limb continued quite well for nine years, and then broke out in the old cicatrix in the early part of 1837, and he was admitted into the hospital in the following December, under my care, when 60 years of age, with a cancerous fungus of about three inches and a half in diameter, attended with much pain. Amputation was proposed to him, as the bone was felt at the bottom of the fungus, but he preferred trying the effect of caustic first, and chloride of zinc was applied to half of the sore. This was followed by inflammation of the absorbents and cellular texture, and much disturbance of the health, and some weeks afterwards I amputated the leg below the knee. The bone even now was only superficially affected, but on the man's death, from inflammation of the veins, I found several of the glands in the groin enlarged with hard firm white substance, with some of the yellowish deposit in it, which is often found in cancerous glands, and which left no doubt in my mind of their being contaminated by the disease of the leg below them; one of them was as large as a walnut, the others were smaller.

This is, however, the only instance I have seen of the absorbent glands being affected, out of perhaps some twenty-five cases of the disease, of which I have either notes or a distinct recollection; and therefore surgical operation for the cure of this kind of cancer can be undertaken with tolerable confidence. The occurrence shows the similarity of the cancer of cicatrices, and of the skin; but at the same time it demonstrates their dissimilarity in degree, as infection in the common cancer of the skin, especially when of such long standing, is undoubtedly in a greater proportion of cases than this. How many glands may be affected by common cancer of the skin, you have seen in Mr. Tatum's case, recently operated on, many of the glands of the neck being in that case enlarged, besides some tubercles in the skin, separate from this fungous ulcer which was removed.

*Thirdly*, as to the general state of the system in cancer of cicatrices. There was no disease found in any part of the body in Callcott, nor in another patient whom I carefully examined after death, with the very large and fatal ulcer which I before mentioned to you; nor, I believe, in Mr. Walker's patient, to whom this limb belonged; nor was there any evidence of local disease, nor of cancerous deterioration of the health, in any of the other patients whom I have operated on, or seen under other surgeon's care. It is probable, therefore, considering the number of cases I have seen, that such an occurrence is extremely rare, and particularly as, even in the more virulent cancerous ulcerations of common skin, the system in very many cases appears not to be contaminated by the local disease. We lost the opportunity, in Mr. Tatum's case, of seeing whether there was any cancerous deposit in the viscera, as, owing to some cause or other, only the head was examined; but I understood there were no symptoms of disease before his death, except in the head, where no morbid deposit was found.

Thus, then, if cancer of cicatrices is so mild in its effects as to be nearly 'always local—if the glands are only affected in some rare case, and the system still more rarely becomes contaminated, although the disease has extended deeply and largely even into the cancelli of the bones—a cure may reasonably be anticipated in almost every case. But then, again, as the disease is not merely an ulcer of unhealthy character, but is essentially a morbid deposit, capable of spreading into the adjacent parts, nothing short of the entire removal or destruction of every portion of the new growth can produce this cure.

There are but two methods of effecting this object, namely, the use of caustics, or of the knife of the surgeon. Each of these may be applicable in some cases; or where both may be employed effectually, the patient may sometimes have his choice of the two plans. For small portions of the disease, or superficial ulcers, caustic may be employed as safely as the knife, although perhaps with more pain to the patient, whose fears often lead him to choose the caustic in preference to the other mode. If the thickness is great, the knife must be much less painful, and probably safer, than the use of caustic.

As a general rule, I think caustic is less likely to be followed by inflammation of the cellular tissue, with risk of absorption of matter, and the formation of secondary abscesses, than an operation by the knife. But then, on the other hand, I think you will much more frequently have inflammation of the absorbent vessels, and erysipelatous inflammation of the skin, after the use of strong caustics, than when a cutting instrument is used: so that I scarcely know which is really attended with more risk to the patient from these accidents. When you employ caustic, it must be one active enough to destroy the whole thickness of the disease; so that the actual cautery, or lunar caustic, are useless; even nitric acid will often extend with difficulty through the new structure. Kali purum, or the chlorides of zinc or antimony, may be employed without the risk which attends the use of arsenic; but I think the best of all is the chloride of zinc, which may be proportioned, with a little experience, to the exact thickness of the part to be destroyed in different cases. Sometimes, then, the tumor or ulcer may thus be taken away completely; sometimes, when a bone is exposed, and rough or carious, a chisel will scrape off the surface, without, in its inflamed state, causing the exposed layer to exfoliate; or, if it is too hard for this, the surface of the bone may be touched by nitric acid, when the bleeding has ceased after the operation, exfoliation of course then taking place. Sometimes, again, the disease is too extensive, or too deep, to admit of removal without amputation of the limb in which it is situated.—*Ibid.*

---

*On the Detection of Arsenic.*—The following important report was read at the Academie des Sciences, June 14th, by M. Regnault:—

The Academy has charged M.M. Thenard, Dumas, Boussaingault, and myself, to give in a report on several memoirs and communications addressed to it concerning the employment of Marsh's apparatus in medico-legal researches. (Here follow the names of five memoirs.)

Before pointing out the results contained in these writings, and before mentioning the experiments which we have made to verify them, it appears to us indispensable to state, as briefly as possible, the state of the question at the time when they were addressed to the Academy. (Here the reporter passes to the origin of Marsh's apparatus, &c., and then enters on the description of the numerous experiments undertaken by the commission in order that it might decide on the important questions referred to it.) These experiments permit the following propositions to be established:—

1. Marsh's plan easily renders sensible $\frac{1}{1000000}$ of arsenious acid in a liquid; spots begin to appear with liquid containing only $\frac{1}{2000000}$

2. The spots are not shown better when a large quantity of liquid is employed in the apparatus than when a smaller quantity is used, provided the same proportional quantity of arsenious acid is present; but they form during a longer period in the first than in the second case. It thence results that there is an advantage in concentrating arsenical solutions, and in operating on a small volume of the liquid; for much more intense spots are thus obtained.

3. It is of the greatest importance, when it is desired to produce spots by means of Marsh's apparatus, to place in the way of the gas a tube three decimetres long filled with amianth or with cotton, in order to catch the little drops of solution which are always carried forward mechanically by the gas; otherwise one is exposed to the danger of obtaining oxi-sulphate of zinc, which often presents the appearances of arsenical spots.

4. The process proposed by M. Lassaigne may produce good results. It consists in making the arseniurretted hydrogen gas pass through an exactly neutral solution of nitrate of silver, then decomposing the liquid by chlor-hydric acid, evaporating it to drive off the acids, and then testing the residue with the arsenic-reagents. It is especially convenient for transferring into a small quantity of the liquid a very small portion of arsenic existing in a large volume of liquid which cannot be concentrated by evaporation, and consequently for permitting one to obtain much more marked arsenical spots by treating the now concentrated arsenical liquid in a very small Marsh's apparatus. Only care must be taken not to decide on the pressence of arsenic, because the solution of nitrate of silver is rendered turbid, and lets fall a deposit during the passage of the gas, since the deposit may be produced by gases not arsenical when mixed with hydrogen, and even by hydrogen alone if the operation is carried on under the influence of light. The solution of nitrate of silver may be replaced by one of chlorine, or of an alkaline chloride.

5. The plan pointed out by MM. Berzelius and Liebig, and variously modified by Kapelin and Kaupmann de Colmar, demonstrates quantities of arsenic which are not shown at all, or only in a doubtful manner, by the spots. This plan therefore presents the advantage of condensing the arsenic in a much more complete manner; only it will frequently happen that the arsenic will be found mixed with sulphuret of arsenic, which may alter its colour, especially if the arsenical substance exists in small quantity.

It is to this last plan that your commissioners give the preference for seperating arsenic. They think that the apparatus should be disposed in the following manner:—A straight-necked and wide-mouthed flask is closed by a cork bored with two holes. Through the first of these holes a straight tube, one centimetre in diameter, is passed down to the bottom of the flask, and in the other is placed a narrow tube bent at a right angle. The end of this tube is placed in another larger, about three decimetres long, filled with amianth. A tube of hardly fusible glass, two or three millimetres in diameter, is adapted to the other end of the amianth tube, and is drawn out at its entrance, and enveloped for a short distance with gold or silver leaf. The flask must be able to contain all the liquid to be tested, and to have still an empty space equal to about one-fifth of its total capacity. It must be remembered, however, that it is important that the volume of the liquid should not be too considerable, if the liquid to be examined be one that contains only traces of arsenical matter. The disengagement tube is terminated *en biseau* at the end which is passed into the flask, and carries a little hollow sphere at some part of its vertical branch. This disposition is not indispensable, but it is convenient, because it condenses almost all the water that is carried up by the gas, and makes it fall back into the flask.

The apparatus being thus disposed, some laminæ of zinc are introduced into the flask, then a layer of water to close the aperture of the safety-tube, and then a little sulphuric acid. The hydrogen which is disengaged drives the air out of the flask. The tube is now heated with a coal fire at the part which is enveloped with gold-leaf; a small screen being interposed to prevent the parts too far off from being heated. When the tube is red hot, the suspected liquid is introduced by the open tnbe through a fine-tubed funnel, so as to make it descend along the walls, and prevent it from carrying air into the flask. If the disengagement of gas is lessened after the introduction of the liquid, a small quantity of sulphuric acid must be introduced, and the operation must be made to go on as slowly and as regularly as possible.

It the gas contains arsenic, it is deposited in the form of a ring in the part of the tube which is anterior to the heated part. The gas which is disengaged from the tube may be set fire to, in order to try and collect spots on a little cover of porcelain; or the tube may be bent, and have its extremity passed into a solution of nitrate of silver, to condense, if necessary, the last portion of arsenic.

The arsenic being deposited in the tube in the form of a ring, it is very easy to determine all the physical and chemical properties which characterize that substance. Thus, one may determine easily, first, its volatility; secondly, its change into a white powder, arsenious acid, when the tube is heated during the passage of a current of air through it; thirdly, by heating a little nitric acid or aqua regia in the tube, the arsenious is made to pass into arsenic acid, which is very soluble in water. The liquid, cautiously evaporated to dryness in a little porcelain capsule, will give a brick red precipitate when some drops of a neutral solution of nitrate of silver are poured into the capsule; and lastly, when all these trials have been made, the arsenic may be again produced in its metallic state. For this purpose, it is sufficient to add a small quantity of black flux in the little capsule in which the precipitation by nitrate of silver has been effected, to dry the mixture, and introduce it into a little tube closed at one end and drawn out at a lamp. By submitting the part of the tube which contains the mixture to a full red heat, the arsenic passes to the metallic state, and forms, in the very narrow part of the tube, a ring which presents all the physical characters of arsenic, even when only very small quantities of that substance exist.

6. It is easy to find, in commerce, zinc and sulphuric acid which do not give any signs of containing arsenic with a Marsh's apparatus, even when considerable quantities of zinc are dissolved. The sulphuric acid which we employed was purified by distillation, and the zinc was laminated zinc in fine slips.

In all cases it is indispensable to test previously, with the greatest care, all the substances to be employed in researches. We even think that some preliminary attempts do not give a sufficient guarantee of security; and that it is necessary for the experimenter to make, at the same time as, or directly after, his examination of the poisoned substances, an exactly similar blank experiment, employing all the same reagents in the same quantities as in the real operation.

7. The processes of carbonization of the animal matter by nitric acid or nitrate of potass may succeed completely; but still it sometimes happens that one cannot prevent a very vivid deflagration at the end of the experiment; and this may give rise to a notable loss of arsenic. The carbonization by concentrated sulphuric acid, and the treatment of the resulting material with nitric acid, or aqua regia, appears to us, in a great number of cases, preferable. This plan, which is suggested by MM. Danger and Flandin, requires the employment of a much smaller quantity of the reagent; it is always easy to conduct, and when properly executed is accompanied by the loss of a very small quantity of arsenic; and even this loss may be avoided by carrying on the carbonization in a glass retort connected with a recipient.

8. It is of the greatest importance that the carbonization of the organic matter should be complete; without this, one obtains not only a liquid which grows mouldy in the Marsh's apparatus, but this liquid may produce spots which sometimes present much resemblance to arsenical spots. These spots, which were first observed by M. Orfila, are often produced in great abundance when the organic matter has been only partially destroyed. They proceed from the carbonaceous gas, partly decomposed in the flame, but are very easily distinguished by chemical reagents from the true arsenical spots. The latter dissolve instantaneously and in the cold in a few drops of nitric acid; the solution, evaporated to drive off the excess of nitric acid, and then treated by neutral nitrate of silver, gives a brick red deposit of arseniate of silver: while the non-arsenical spots dissolve with more difficulty in nitric acid, and there always remain some particles of brown matter which do not disappear till the acid is heated; and when all is dissolved, the solution, evaporated to dryness, and treated by nitrate of silver, gives a yellow deposit of phosphate of silver. Thus nothing is more easy than to distinguish these from the true arsenical spots. If the latter are themselves mixed with foreign matters, as happens when the carbonization of poisoned food has been imperfect, then these characters become less striking; but these spots may give rise to very serious mistakes if the experimenter contents himself with physical characters which resemble one another.

9. As to the arsenic which has been announced to exist in the healthy human body, all the experiments which we have made, both on the muscles and on the bones (and on soup) *have given us negative results.*

10. The commission, therefore, thinks that Marsh's method, applied with all the precautions that have been pointed out, is sufficient for medico-legal researches, in which the quantities of arsenic which it is required to detect are almost always much greater than those of which the sensibility of this apparatus enables us to determine the existence. Understanding clearly, however, that it must always be employed as a *means of concentrating* the metal for the purpose of studying its chemical characters, and that one must consider as *null, or at least as very doubtful*, the indiactions which it will furnish, if the deposit which is formed in the anterior part of the tube does not permit the examiner, on account of its slight thickness, to verify, in a precise manner, the chemical characters of arsenic.—*Ibid.*

---

*On the Cure of Cretinism.* By Dr. Guggenbuhl.—To diminish the considerable number of cretins in the narrow valleys of the Alps, an establishment has been formed for the education of children who, in their infancy, present indications of this condition. It has long been ascertained that all young cretins might be cured by transferring them from their low valleys to the high mountains; and that at a distance of about 3000 feet above the level of the sea these unhappy creatures are no longer met with. All the midwives of the narrow valleys easily recognise cretinism at the time of birth: they know that every child born with a stupid expression of face, uttering peculiar cries, and making strange movements, is sure to be a cretin.—*Gazette Medicale*, Mai 8. 1841, *from Haeser's Archiv. für die Gesammte Medicin.*

